# Yellow or Bulam Fever

**Published:** 1817-01

**Authors:** 


### YELLOW OR BULAM FEVER.

As the medical public must be anxious to learn the result of the important question relative to yellow or Bulam fever, now agitated by government, we have much pleasure in being able to bring forward the following report, one of the most able and interesting documents which have been transmitted to government from the naval medical corps. We have reason to believe that it speaks the sentiments nearly of that whole body. EDITORS.

To Alexander M'Leay, Esq. &c. &c. &c.

Naval Hospital, Barbadoes,

January 1, 1816.

Sir,

Having been called upon by the Board of Commissioners, to furnish them with all the facts that may have come to my knowledge, either in support or in refutation of an opinion published by Doctor Pym, that the human frame is not liable to receive the infection of the "Bulam (commonly called Yellow) Fever" twice; and to add to those facts any remarks that may suggest themselves to me; and also previously to complying with this request, to transmit, by the first opportunity, a reference to any medical gentleman, whose experience and whose journals might afford farther information:

I have already, in obedience thereto, cited several whose employments during the prevalence of fever in this country, have given them opportunities of seeing it in its various forms of mildness and malignity, and of distinguishing the difference (if any there be) between the fevers of marsh miasmata, and those arising from the evolution of foul air on board ship; or from other causes eluding detection.

In proceeding to relate facts that have occurred in my own practice in the West Indies, since the early part of 1807, it may be well to premise that many of them may by the unprejudiced be deemed conclusive; shewing that the fevers which at different periods appeared amongst us, and at particular seasons proved so destructive to the newly arrived Europeans, may be referred exclusively to the action of miasmata; that in no instance have they been proved infectious; and that, though the human frame seldom experiences a second attack of the disease, it is by no means exempt from its recurrence.

Dr. Pym has endeavoured to establish a distinction between "bilious or yellow fever, and that disease which is said to have been imported into Grenada from Bulam, the former attacking the same individual again and again; the latter highly contagious and communicable, to be prevented only by a strict adherence to the laws of quarantine, and rendering the constitution illiable to future invasion."

Having held the several hospitals at Mariegalante, Antigua, Martinique, and Barbadoes, during periods when fever was epidemic at those islands, many opportunities have been afforded me of observing it in its various modifications, and in several instances of detecting its origin. But from whatever causes arising, I have seen during the years 1808, 1809, 1810, and part of 1811, the same train of symptoms, the same suddenness of invasion, and rapidity of progress to recovery or death common to all. I have never during these periods had occasion to think, that the disease was communicable from one individual to another, though it has prevailed generally on board, and in some of the garrisons.

I have, however, repeatedly known individuals who having survived one attack, have on a second fallen victims; the same violent symptoms recurring, and accompanying the sufferers to the close of life.

These instances became first known to me at Mariegalante, where our troops (composed for the greater part of marines) debarked in excellent health, in July 1808.

It being the intention of Government to keep possession of that island, the town of Grandbourg became head quarters, and barracks the most airy and commodious were selected; whatever was deemed necessary to the preservation of the health and the discipline of the garrison was immediately carried into effect by the Governor, Captain Pigot. Nevertheless, but few weeks elapsed before fever, in full malignity, made its appearance; to be attacked, and to fall its victim, were terms almost synonymous.

In consequence of this sudden and increasing mortality affecting considerably the strength of the medical staff, Sir Alexander Cochrane proceeded thither, to devise, with the assistance of the physician to the Fleet, such measures as might appear most necessary, at least to arrest the farther progress of disease. Other barracks in a more elevated situation were chosen, and occupied; and at the particular request of the Commander in Chief, I took the medical charge.

On getting on shore about noon, I came up with several black pioneers, who having found a sailor in the streets, had been called upon to take him to the hospital. The poor man's resistance to their endeavours, his cries, and delirious rage, led me to remonstrate; when they made answer, "Lord, Sir! he has the fever, and will soon be dead!" This prediction was verified at midnight; the delirium continuing to within an hour of his decease, and the vomiting of dark coffee-coloured fluid almost incessant.

Such was my initiation to the duties I had just been called upon to execute. The scenes that presented themselves in the different receptacles for the sick, were indeed shocking: the man invaded; the delirious sufferer; the anxious countenance, very well described in Dr. Pym's publication, yielding up life in expiring efforts to vomit; these were sights occurring day after day, defying every effort of the medical officer, whether of the prophylactic or curative kind.

I suppose it unnecessary to enter into a detail of all the symptoms; it may be deemed sufficient to state, that the progress was, in many instances, so rapid, that men bearing arms in the morning, have in a very few hours been buried in the same grave!

That every facility might be afforded on this distressing occasion, Sir Alexander Cochrane permitted my taking with me two assistant surgeons, and two men who understood the composition of medicines. The senior assistant and one of the compounders were carried off in the first ten days. The junior (Mr. Griffiths), and the other compounder, were lying in the same room with me, extremely low from the loss of large quantities of blood abstracted on the preceding day; when I was myself attacked at 3 A. M. with excruciating pain in the orbits, forehead, and loins. I had no power to use my lower extremities. Thus situated, Mr Griffiths was compelled to quit his cot, and with a trembling hand to put a lancet into my arm, in which effort he sunk on the floor. I felt relief with the flowing of blood, restrained only by the supervention of syncope. Pain soon after recurring, the orifice was re-opened, and freedom given to my oppressed brain:—my recovery was rapid.

In intenseness and malignity, this fever has not been exceeded; and of its resistance to every curative measure (when fully formed) the shocking mortality has borne ample evidence.

Of its recurrence in the same individual, there were many instances, as well among those continuing in the town, as among others removed to different situations in the island. Were not these instances in the recollection of Captain Pigot (the Governor), and several other residents, I could particularize cases in confirmation of my statement.

The town of Grandbourg has extensive swamps on three of its sides; and from the unfavourable effects of the war upon the industry of the inhabitants, there was much uncultivated ground throughout the island.

With the wet season commenced the action of miasma on the troops. For the first fortnight, no men enjoyed better health; during that time, much rain fell, and great heats succeeded. Need we more fruitful sources of disease?

In the rise, progress, and too general termination, of this malady, it bore an intimate resemblance to that so correctly described by Dr. Chisholm. The seizures were almost universal amongst the newly arrived occupants (for all were equally exposed to the causes which produced it) but that it was in any instance communicated from one person to another, I had no reason to suspect.

The settled inhabitants generally enjoyed good health, (with the exception of intermittents) as did several people recently from Guadaloupe.

It may here be proper to say something of the various modes of treatment pursued during the prevalence of this epidemic. Fever once formed mocked depletion by the lancet, however profusely employed. Equally inert were calomel and mercurial innunction, in whatever large quantities administered or employed. Purging had as little effect. It became a serious object to arrest (if possible) the *formation* of disease; for which purpose the Governor, with his usual kindness, permitted me to examine the men, when drawn up upon the parade. The aberration from a state of health was easily discovered in the altered appearance of the eye, and in the dejected countenance. The former had a brown, muddy, watery appearance; on enquiry, it was found, that more or less of stupor, or vertigo, was experienced. The pulse was generally slow, and full; and the alvine dejections scanty and costive; with such symptoms of approaching illness, the man was immediately sent to the bleeding room, and blood abstracted, not by measurement, but until syncope was sometimes twice produced. Succeeding to this, calomel, combined with a resinous purgative, or the sulphate of magnesia, was given to free the belly of its vitiated contents; after which, bark in doses of ʒj secundâ quaque horâ.

The number of severe and fatal attacks soon diminished, and at the early period of my quitting to rejoin the flag, returning health was apparent.

Had this practice been resorted to at the onset, the success which attended it *when* adopted, leads me to think mortality would have been much less considerable.

Should I ever again be witness to epidemic fever, in a warm climate, in the same degree of malignity, my advice would be to mark most attentively the least aberration from a state of health, and at the instant, to release the blood vessels of a large quantity of their contents.

Since the early part of 1811, the fever so long the scourge of this country, has nearly ceased to be fatal; the "Bulam" (as Dr. Pym designates it) has been seldom witnessed; and the West Indies, generally, have enjoyed a degree of health not exceeded by any country in the world.

But during many years of its epidemic influence, no island was perfectly exempt. In some the mortality was uncommonly great, and, amongst these, English Harbour at Antigua claims particular attention.

On a reference to the returns made regularly to Dr. Dickson, then physician to the fleet, we shall find that the number of men attacked at English Harbour, whilst their vessels were there under repair, exceeded all the sick of the West India most numerous fleet, (a fleet consisting of eighty pendants) and not only in number, but in severity, and like "Mariegalante" in dreadful mortality.

Well indeed might that able physician, a spectator of the scenes he describes, thus express himself.

"Could a doubt exist, as to the fatal powers in operation at that depôt, it would be removed by a review of the number and mortality of fevers during the last quarter.

"It appears, that in the months of October, November, and December, there were in the Naval Hospital, seven hundred and fifty-four cases of disease; of which, six hundred and twenty-two were fevers; and, that of this number, one hundred and ninety-seven died. In December alone, it contained three hundred and sixty fevers, whereof one hundred and forty-nine were discharged, and one hundred and thirty-three baffled the efforts of medicine, evincing the very great mortal tendency of this malady.

"This will receive further elucidation by references to a few particular ships."

"The Paerlin, before and after the commencement of the quarter, was visited so severely by sickness, that according to the report of the surgeon, "the diminution of disease seemed only to be influenced by the diminution of numbers." The Thetis, in November, sent one hundred and eighty-two to the hospital, of whom eighty-four died.

"The Nyaden, about the same time, lost thirty-three out of ninety-eight, sent to the same place; and immediately afterwards, fourteen on board, and ten out of thirty-four sent to the hospital on arriving at Barbadoes; nor has that ship yet ceased to feel the effects of having refitted at English Harbour.

"But the small vessels are still more strongly illustrative of the malignant powers existing there, because from having been in that harbour before, and from having been a considerable time in the country, they must be better steeled against disease.

"Yet out of twenty men, constituting nearly the whole of the crew of the Grouper sent to the hospital, eight died; of thirty-four from the Bacchus, eleven died; of seventeen from the Forrester, six died; of nineteen from the Demarary, ten died; of fourteen from the Laura, seven died; and of six from the Balahou, three died:—proving (these with the exception of the Forrester and Demarary brigs, being schooners with very few men) how generally sickness must have prevailed; and proving also, in my opinion, incontrovertibly, how incompetent is the human frame, when prepared for fever by excitement and irregularities, to resist its attacks; and hence, how frequently fatal must be the consequence of refitting at Antigua."

Such were the attacks and results of fever amongst the shipping; nor, during this period, did the inhabitants residing in certain situations, in the vicinity of English Harbour, escape its influence. I believe that several born near the spot fell victims to the malady. The family of the Dispenser of the Naval Hospital suffered most severely: in three weeks he lost his mother in-law, her son, and his two children, all natives of the country. The Dispenser and his wife were both seized, and the latter recovered unexpectedly.

In 1808, a similar visitation attended Dr. Ralph Cumming and his family; Mrs. C was carried off first, and on the day week of her decease, were buried, in the same grave, the Doctor and his child.

The Doctor's successor was not more fortunate, though he had served five years as surgeon of the Jason frigate, without quitting the country.

Mr. Sampson Hardy (the gentleman alluded to) took charge of the establishment the latter end of July, and fell a victim to fever on the last day of August.

In the following October, I was ordered to succeed him; at this time, there was not a case of fever either in the hospital or in the harbour. It soon after recurred, and although I had suffered so recently at Mariegalante, I was again to experience the full force of its malign influence.

I had every bad symptom, (save black vomit) and my recovery was deemed next to impossible by those in attendance.

It has appeared highly necessary to relate, somewhat at large, a few of the circumstances occurring at this Depôt; they assist to shew the great malignity of the fever, the almost universal seizures of the crews, and its rapidly mortal tendency.

On my entering on duties so infinitely important, I was anxious to learn of the practitioners of the island all the facts they had become acquainted with respecting this malady; for which purpose the following letter and queries were sent to them.

" *Circular.*"

" SIR,

Naval Hospital, Antigua, 1809.

" Being anxious to collect as many facts as possible relative to the endemial fever of the country, I am well assured you will pardon the liberty I am about to take, in entreating your opinion on the following heads:—Your extensive practice and matured experience give to you the ability of determining, in some degree, the influence of causes which, probably, if better understood, would lead to greater exemption from seizure, and to a more successful practice than has hitherto obtained.

" Improvements in the mode of treatment have certainly taken place, if we may judge from the general issue; and perhaps, there only requires a more frequent communication of successful practice to disarm this fatal disease of much of its dreadful malignity.

" I am, &c.

" J. M."

Query 1st.—What are the most common causes of endemial fever?

2d.—How is it best characterized?

3d.—In what months most prevalent and malignant, and in what most mild?

4th.—Is the production and character of the disease influenced by particular viands? If so, what are they? and why?

5th.—The best mode of treating this disease?

6th.—Is the endemia a fever sui generis?

7th.—Much benefit having been derived from the application of cold to the surface, has spirit (commonly called rum) any effect superior to watery affusion?

The topography of English Harbour and its environs leave no doubt in a medical man, that without increasing exertion, causes believed by all to be sufficient for the production of fever any where, will be found there in the greatest abundance.

With an outlet into the sea not wider than the length of two line of battle ships, with a most extensive swamp directly to windward, and much of still water leading to the mast-pond to the northward, into which it was for years the custom of the ordnance department to empty all their exuviæ; and bounded (with the exception of the before-mentioned opening) by high hills; it is to be expected of such a land-locked situation, that fever, when epidemic, shall always prevail there in great malignity.

My earnest wish is to state facts without bias to any particular opinions; and for the correctness of my statement, not only of the fever, but of the probable causes leading to it, I would refer to the experience and accurate judgement of Dr. James Veitch, who held the naval hospital at the time Dr. Dickson wrote his letter. I would refer also to his coadjutor Mr. Sheppard, from whose indefatigable disposition for research, much soundness of opinion may be expected.

It is worthy of remark, that whilst fever has so repeatedly thinned the crews of vessels refitting at English Harbour; a bay contiguous to it, named Falmouth Bay, has been perfectly exempt from the causes which would seem to be so generally obnoxious to the human frame in the former place.

It becomes me here to pay a tribute of well-earned praise to the present superintendant of the dock-yard at Antigua. To his unwearied industry and skill, and to his unceasing exertions, may justly be ascribed the late greater exemptions from fatal fever at that important depôt. The Swamp (I understand) has been drained; the throwing the accumulated filth of the ordnance department into the mast pond interdicted; and generally, such orders and regulations enforced, as have assuredly had no small influence in rendering the refitting at English Harbour much less inimical to life, and almost innocuous to health.

I have not had the advantage of perusing Dr. Bancroft's publication. He is stated by Dr. Pym to deny the powers of offensive vapours emitted by matters in a state of decomposition to produce fever; but as I have already observed, that in some instances I had detected the origin of this disease, and by its removal restored health, I will now relate some of them as illustrative of this subject.

The Nyaden frigate quitted Carlisle Bay to refit at English Harbour, with her crew in ordinary health. Her services being much required, she was ordered to wood and water at Dominica, and to proceed, with all due dispatch, to Antigua. I had ascertained that the wood was taken on board in a green state; and after the ship had lost one-third of her crew, partly where she refitted and partly on her passage thence to Barbadoes, Sir Francis Laforey directed me to report to him the probable cause of this coutinued mortality. I ascribed it greatly to the taking in of this green wood, and recommended her complete clearance and exposure to fumigation. No time was lost in carrying these steps into execution, as directed by the commander in chief; and the consequences were immediate and most happy. But very few attacks succeeded, and those unattended by fatality.

So far as my own observations have gone, the appearance and progress of fever have depended on localities; and I have been satisfied in the greater number of instances, where it has prevailed on board particular ships, that to some such causes as above alluded to it might be ascribed.

( To be continued. )

